# Could the Adoptive Transfer of Memory Lymphocytes be an Alternative Treatment for *Acinetobacter baumannii* Infections?

**DOI:** 10.3390/ijms251910550

**Published:** 2024-09-30

**Authors:** Tania Cebrero-Cangueiro, Soraya Herrera-Espejo, María Paniagua, Gema Labrador-Herrera, José Miguel Cisneros, Jerónimo Pachón, María Eugenia Pachón-Ibáñez

**Affiliations:** 1 Clinical Unit of Infectious Diseases, Microbiology and Parasitology, Institute of Biomedicine of Seville (IBiS), Virgen del Rocio University Hospital/CSIC/University of Seville, 41013 Seville, Spain; tcebrero-ibis@us.es (T.C.-C.); sherrera-ibis@us.es (S.H.-E.); maria.paniagua.sspa@juntadeandalucia.es (M.P.); gemalabrador@hotmail.com (G.L.-H.); josem.cisneros.sspa@juntadeandalucia.es (J.M.C.); 2 CIBER de Enfermedades Infecciosas (CIBERINFEC), Instituto de Salud Carlos III, 28029 Madrid, Spain; 3 Department of Medicine, School of Medicine, University of Seville, 41004 Seville, Spain; pachon@us.es; 4 Institute of Biomedicine of Seville (IBiS), Virgen del Rocio University Hospital/CSIC/University of Seville, 41013 Seville, Spain

**Keywords:** memory lymphocytes, murine pneumonia model, *Acinetobacter baumannii*

## Abstract

We evaluated the efficacy of the adoptive transfer of memory B, CD4+, and CD8+ T lymphocytes compared with sulbactam and tigecycline in an experimental murine pneumonia model by two multidrug-resistant *Acinetobacter baumannii* strains, colistin-susceptible AbCS01 and colistin-resistant AbCR17. Pharmacodynamically optimized antimicrobial dosages were administered for 72 h, and intravenous administration of 2 × 10^6^ of each of the memory cells in a single dose 30 min post-infection. Bacterial lung and blood counts and mortality rates were analyzed. Results showed that a single dose of memory B or CD4+ T cells was as effective as sulbactam in terms of bacterial clearance from the lungs and blood compared with the untreated mice or the tigecycline-treated mice inoculated with the AbCS01 strain. In the pneumonia model by AbCR17, a single dose of memory B or CD4+ T cells also reduced the bacterial load in the lungs compared with both antibiotic groups and was more efficacious than tigecycline in terms of blood clearance. Regarding survival, the adoptive transfer of memory B or CD4+ T cells was as effective as three days of sulbactam treatment for both strains. These data suggest that adoptive memory cell transfer could be a new effective treatment of multidrug-resistant *A. baumannii* infections.

## 1. Introduction

Infections caused by *Acinetobacter baumannii* are mainly healthcare-associated, such as pneumonia, bloodstream infections (BSIs), urinary tract, skin, and other soft tissues, and, less frequently, community-acquired infections [1]. The emergence and persistence of multidrug-resistant (MDR) infections, including carbapenem-resistant *A. baumannii* (CRAB), pose a difficult global challenge. Drug resistance, virulence, and scarce treatment options have led to severe nosocomial infections, especially among intensive care and vulnerable patients, with an alarmingly high mortality rate. In a systematic analysis in which the authors estimated deaths and disability-adjusted life years attributable to and associated with bacterial antimicrobial resistance (AMR) for 23 pathogens in 204 countries and territories in 2019, they found that six pathogens were responsible for more than 250,000 deaths associated with AMR, including CRAB. Together, these six pathogens were responsible for 929,000 (95% UI: 660,000–1,270,000) of 1.27 million (95% UI: 0.911–1.71) deaths attributable to bacterial AMR [2]. Moreover, CRAB is one of the five top pathogens worldwide in terms of attributable mortality caused by antibiotic-resistant infections and is estimated to be the leading pathogen in Southeast Asia, East Asia, and Oceania for mortality attributable to MDR pathogens [3,4].

In May 2024, the World Health Organization published an update to the list of the most dangerous drug-resistant bacteria to human health [5], first developed in 2017, in which *A. baumannii* remained a critical priority pathogen because of its ability to transfer resistance genes and the severity of the infections and disease it causes and/or their significant global burden. However, since the classification of *A. baumannii* as a critical pathogen in 2017, no new drug effective against *A. baumannii* MDR strains has been introduced, emphasizing the persistent challenge and the crucial need for ongoing investment in R&D [6,7]. Nevertheless, despite the urgency, antibiotic development has been lagging in addressing this challenge. 

Immunotherapy has shown efficacy in clinical settings for the treatment of other pathologies, such as cancer [8]. Additionally, the memory lymphocyte transfer approach has been used for neurodegenerative disorders [9] and viral infections [10]. In the case of MDR bacterial infections, there are limited studies. However, most of the studies on Gram-negative MDR bacilli infections are focused on cytokine production and modulation [11,12]. Some studies have aimed to develop protective immunity against bacterial infections through the adoptive transfer of human serum against invasive staphylococcal disease in a murine model [13] or cells, such as immortal phagocytes, to treat neutropenic mice infected with *Staphylococcus aureus*, *A. baumannii*, *Candida albicans*, or *Aspergillus fumigatus* [14]. Regarding innate immunity, monocyte-derived macrophage cell transfer was able to confer protection in murine peritonitis models infected with *Klebsiella pneumoniae* and methicillin-resistant *S. aureus* (MRSA), as well as in lung infection models infected with *Pseudomonas aeruginosa* [15]. Similar results were documented in meningitis or cutaneous infection mouse and rat models infected with MRSA or *A. baumannii* and treated with bone marrow-divided macrophages [16]. Regarding adaptive immunity, the concept of adoptive cell transfer has its roots in cancer immunotherapy, where it has achieved significant success [8]. Memory lymphocytes (B, CD4+, and CD8+ T cells) play a crucial role in the adaptive immune system due to their fast and robust ability to respond upon re-exposure to specific pathogens. Dunkley et al. [17] concluded that CD4+ T transfer from previously immunized donors to *P. aeruginosa*-infected rats enhanced bacterial clearance from the respiratory tract. Furthermore, bacterial clearance was higher when antigen-specific T lymphocytes were used [17]. 

Therefore, in the present study, we aimed to evaluate a novel approach using the adoptive transfer of memory lymphocytes to treat infections of *A. baumannii* strains with different susceptibility phenotypes in a murine pneumonia model in an attempt to diminish the mortality and tissue and blood bacterial loads associated with these strains. 

## 2. Results

### 2.1. Characterization of Test Strains

#### 2.1.1. Surface Motility Assay

**Figure 1:**
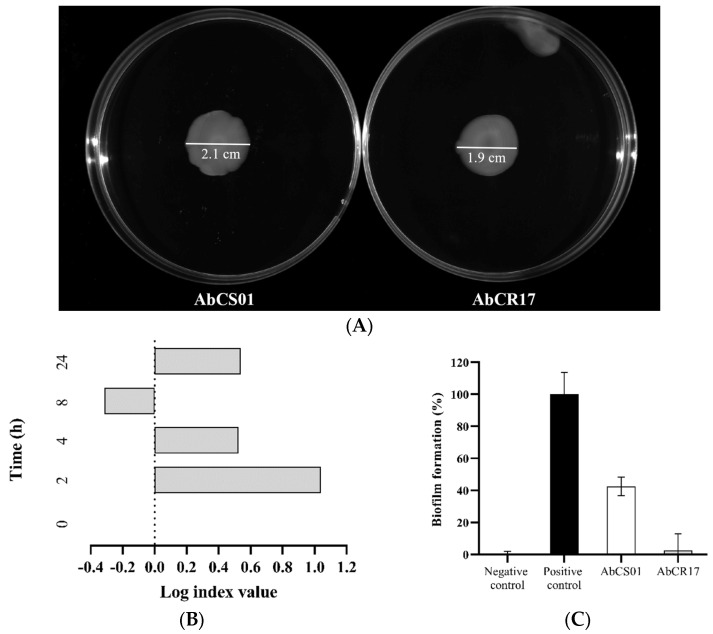
In vitro characterization of test strains (surface motility, competition index (CI), and biofilm formation) of the *Acinetobacter baumannii* AbCS01 and *A. baumannii* AbCR17 clinical strains. (**A**) Surface motility of the AbCS01 and AbCR17 clinical strains; (**B**) CI. Each bar in the graph represents the mean values of the Log index. A CI value equal to zero indicates no competition between the two species; a positive CI value indicates a competitive advantage for AbCR17, and a negative CI value indicates a competitive advantage for AbCS01. (**C**) Biofilm production of both clinical *A. baumannii* strains. Dark gray bar: negative control (*A. baumannii* AC001); black bar: positive control (*A. baumannii* ATCC19606); white bar: AbCS01; gray bar: AbCR17.

No difference was observed in surface motility between the AbCS01 and AbCR17 *A. baumannii* strains (Figure 1A).

#### 2.1.2. In Vitro Growth Curves and Competition Indices (CI)

When studied alone, both strains, AbCS01 and AbCR17, showed almost identical growth kinetics. However, in competition, the colistin-susceptible strain, AbCS01, showed a growth disadvantage over the colistin-resistant AbCR17 strain (Figure 1B).

#### 2.1.3. Biofilm Assay

The colistin-susceptible AbCS01 and colistin-resistant AbCR17 strains showed 60% and 95% less biofilm formation, respectively, than the reference strain *A. baumannii* ATCC 19606 (positive biofilm control). No significant differences (*p* = 0.100, Mann–Whitney U test) in biofilm production were found between the two tested *A. baumannii* strains (Figure 1C).

### 2.2. In Vivo Studies

#### Efficacy Studies in a Pneumonia Murine Model Infected with *A. baumannii* AbCS01 and *A. baumannii* AbCR17 Clinical Strains

**Figure 2:**
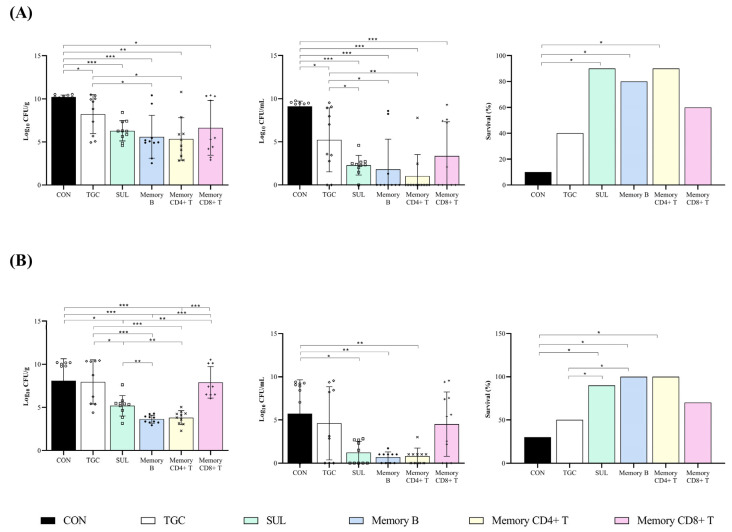
Bacterial concentrations in the lungs and blood and survival rates after treatment with antibiotics and the adoptive transfer of memory B and T lymphocytes in the pneumonia model with multidrug-resistant (MDR) *A. baumannii* CS01 and CR17 strains. (**A**) Bacterial concentrations in the lungs (**left**) and blood (**medium**) and survival rates (**right**) of mice infected with AbCS01; (**B**) bacterial concentrations in the lungs (**left**) and blood (**medium**) and survival rates (**right**) of mice infected with AbCR17. CON: untreated control group; TGC: tigecycline-treated group; SUL: sulbactam-treated group; Memory B: memory B lymphocyte-treated group; Memory CD4+ T: memory CD4+ T lymphocyte-treated group; Memory CD8+ T: memory CD8+ T lymphocyte-treated group. *: *p* < 0.05; **: *p* < 0.01; ***: *p* < 0.001.

In monotherapy studies to interrogate the colistin-susceptible AbCS01 strain, both antibiotics tested, tigecycline and sulbactam, improved the bacterial clearance from the lungs and blood with respect to the untreated control mice (−1.98 and −3.92 log_10_ CFU/g and −3.87 and −7.91 log_10_ CFU/mL, *p* < 0.05), although sulbactam was better than tigecycline in reducing the bacterial load in blood (−4.04 log_10_ CFU/mL, *p* < 0.05). As for the treatment with a single dose of memory lymphocytes, CD4+ and CD8+ T and B cells decreased the bacterial load in the lungs and blood compared with that in the untreated control animals (−4.86, −3.57, and −4.62 log_10_ CFU/g and −8.08, −5.75, and −7.29 log_10_ CFU/mL, *p* < 0.05). In addition, memory CD4+ T and B cells were better than tigecycline monotherapy at reducing the bacterial load in the lungs (−2.88 and −1.59 log_10_ CFU/g, *p* < 0.05). Moreover, as with sulbactam monotherapy, memory CD4+ T and B cells reduced the blood bacterial load compared with tigecycline monotherapy (−4.21 and −3.42 log_10_ CFU/mL, *p* < 0.05). Finally, both sulbactam and memory CD4+ T cells improved the clearance of blood bacterial load relative to memory CD8+ T cells (−2.16 and −3.42 log_10_ CFU/mL, *p* < 0.05) (Table 1 and Figure 2). Furthermore, in the pneumonia model infected with the colistin-susceptible AbCS01 strain, both sulbactam therapy and treatment with memory CD4+ T and B cells increased survival rates compared with the untreated control animals (+80%, +70%, and +80%, *p* < 0.05) (Table 1 and Figure 2A).

The results of the pneumonia model with the colistin-resistant AbCR17 clinical strain showed that, of the antibiotics used, only sulbactam was able to decrease the bacterial load in the lungs and blood when compared with the untreated control animals (−2.89 log_10_ CFU/g and −4.65 log_10_ CFU/mL, *p* < 0.05). Moreover, treatment with a single dose of memory CD4+ and B cells improved the clearance of bacterial load in the lungs and blood when compared with the untreated control animals (−4.27 and −4.45 log_10_ CFU/g and −5.04 and −5.17 log_10_ CFU/mL, *p* < 0.05). In addition, sulbactam and both memory CD4+ T and B cells reduced lung bacterial load when compared with tigecycline (−2.77, −4.15, and −4.33 log_10_ CFU/g, *p* < 0.05) and memory CD8+ T cells (−2.48, −3.86, and −4.04 log_10_ CFU/g, *p* < 0.05). Finally, the treatment with a single dose of memory CD4+ T and B cells was able to diminish the lung bacterial load in animals infected with the colistin-resistant AbCR17 when compared with sulbactam (−1.58 and −1.56 log_10_ CFU/g, *p* < 0.05) (Table 2 and Figure 2B). Regarding the survival analysis, sulbactam and memory CD4+ T and B cells increased the survival rates with respect to the untreated mice infected with the colistin-resistant AbCR17 strain (+60%, +70%, and +70%, *p* < 0.05) (Figure 2B).

## 3. Discussion

Our results showed that sulbactam monotherapy was better than tigecycline monotherapy in treating experimental murine pneumonia caused by both strains of *A. baumannii*, regardless of the MIC. In addition, a single dose of memory B or CD4+ T cells was as effective as the three-day sulbactam treatment in terms of clearance of the bacterial load in the lungs and blood compared with untreated control mice or the tigecycline-treated (72 h) mouse group against the colistin-susceptible *A. baumannii* strain. In the colistin-resistant pneumonia model, treatment with a single dose of memory B or CD4+ T cells also reduced the bacterial load in the lungs compared with the tigecycline and sulbactam groups and was better than the tigecycline group in terms of blood clearance. With respect to increased survival, treatment with a single dose of memory B or CD4+ T cells was as effective as a three-day treatment with sulbactam at pharmacodynamically optimized doses in the pneumonia murine model for both strains. 

Based on these results, we found that the intravenous (iv) administration of a single dose of memory B or CD4+ T cells was able to reduce the bacterial load in the lungs against both strains when compared with the control group. It is known that memory B and T cells induced by a previous infection provide rapid and effective protective immunity against reinfection or infection. Although most adoptive transfer studies with memory B and T cells are focused on viral infections [18], some studies evaluate the protective effect of CD4+ T cells in bacterial infections. In this sense, the study of Wilk et al. [19] showed that adoptive transfer of lung memory CD4+ T cells conferred protection in naïve C57BL/6 mice against a respiratory infection caused by *Bordetella pertussis*. In another study [20], the iv adoptive transfer of lung CD4+ T cells into naïve C57BL/6 mice prior to challenge resulted in a significant reduction in the bacterial load in the lungs and spleen 24 h post-infection with a mucoid hypervirulent *K. pneumoniae* KP-396 strain. Moreover, in another study of a pneumonia model in rats, the recipients of purified T cells, or CD4+ T cells, from rats previously challenged with *P. aeruginosa* exhibited improved clearance of bacterial load from the airways compared with recipients of cells from unimmunized donors [17]. This protective effect observed with memory CD4+ T cells is probably due to the key role played by the IFN-γ produced by activated CD4+ T cells (Th1 subpopulation) in macrophage activation, explaining the good results found in the efficacy of memory CD4+ T cells in the treatment of experimental pneumonia caused by *A. baumannii*. To the best of our knowledge, although the adoptive transfer of B cells is widely evaluated for viral infections [18,21], fungal infections [22], and other pathologies [23,24], there have been no studies to evaluate the efficacy of memory B cells for bacterial infections. However, we found a study that evaluated the role of B cells in host immune responses to pneumococcal infection. In this study, hepatic B cell transfer markedly increased plasma levels of IgG2a and IgG2b specific to the pneumococcal surface protein A, as well as IgG3 for pneumococcal polysaccharide in recipient mice. Furthermore, when B cells were cultured in vitro with splenocyte CD4+ T cells from mice killed by *Streptococcus pneumoniae* infection, IL-2 production and CD4+ T cell proliferation were observed, suggesting that B cells participated in acquired immune responses by presenting derived peptides to CD4+ T cells [25]. 

In this study, we also evaluated the efficacy of memory CD8+ T cells and found that they were able to significantly reduce the bacterial load in the lungs against the colistin-susceptible AbCS01 strain. CD8+ T cells are mainly effective in phagocytosing/destroying virus-infected cells [26] and tumor cells [27]. Although *A. baumannii* is defined as an extracellular bacillus, based on previous studies in our group showing that *A. baumannii* can invade and cause the death of lung epithelial cells [28], we evaluated whether these cells that phagocyte intracellular pathogens were able to phagocyte these bacteria, as our results confirm. Treatment with memory CD8+ T cells behaved similarly to treatment with tigecycline, reducing the bacterial load in tissues and blood against the colistin-susceptible strain.

The tigecycline dosage used in this study has been proven to be effective in several murine studies using clinical MDR *A. baumannii* strains susceptible to tigecycline (minimum inhibitory concentration (MIC) = 0.5 mg/L) [29]. In the present study, for the colistin-susceptible AbCS01 strain with a tigecycline MIC of ≤0.50 mg/L, monotherapy with tigecycline was effective in clearing the bacteria from the lungs and blood compared with untreated mice. Nevertheless, when used against the colistin-resistant AbCR17 strain, tigecycline was not effective in clearing the bacteria from the lungs and blood or in reducing the mortality rate. It has been reported that by achieving an AUC_0–24_/MIC of ≥4.5 mg/L [30], the tigecycline treatment success rate was >90% for bacteria with a MIC of ≤1 mg/L, explaining the difference in efficacy with this monotherapy against both studied strains. 

Bhavnani et al. [31] detailed that sulbactam concentrations above the MIC (TΔMIC ratio) of 24.5%, 29.3%, and 37.3% were associated with 1, 2, and 3 log_10_ reductions in CFU, respectively, in an experimental lung model infected with *A. baumannii* with a sulbactam MIC of 0.5 mg/L. However, although these ratios were not achieved in this study, the dosage and regime used were able to reduce the bacterial concentration in the lungs and blood and increase the survival rate in the experimental pneumonia infections caused by both the AbCS01 (sulbactam MIC = 4) and AbCR17 (sulbactam MIC = 2) strains. These positive results in terms of bacterial clearance from tissue and blood and increasing the survival rate have also been reported in several studies on sepsis animal models [32,33]. 

This study has several limitations. Firstly, the 3R rules [34] prevent us from increasing the number of strains to confirm the present results; however, we tried to avoid strain-dependent results by choosing two different strains in terms of colistin susceptibility and fitness. Secondly, as in any experimental animal model, there is a general caution against translating preclinical studies to the clinical setting. 

In summary, these data suggest that a single dose of memory B and CD4+ T cells could improve the treatment of MDR *A. baumannii* infections involving either colistin-susceptible or colistin-resistant strains. 

The promising results of this study will be further investigated in female murine models in a future study. Further studies are necessary to evaluate the potential for combined memory cell and antibiotic therapy to enhance the efficacy of antibiotics. If this hypothesis is confirmed, the next step would be to perform randomized controlled clinical trials to prove whether this novel approach improves the outcome of infections caused by MDR *A. baumannii* strains. Thus, we will evaluate whether the combined treatment of memory B or CD4+ T cells with antibiotics improves the treatment of pulmonary infections caused by MDR *A. baumannii* strains. 

## 4. Materials and Methods

### 4.1. Antibiotics

For the in vitro studies, standard laboratory powders of antimicrobials were used (Sigma-Aldrich, Madrid, Spain). For in vivo studies, the following clinical formulations were used: tigecycline (TGC, Tygacil 50 mg, Pfizer, Madrid, Spain) and sulbactam (SB, Betamaz 1 g, Group Farmasierra laboratory S.L, Madrid, Spain).

### 4.2. Bacterial Strains 

Two MDR *A. baumannii* isogenic clinical strains, AbCS01 and AbCR17, were used, which were isolated from the cerebrospinal fluid of a patient with meningitis [35]. The antibiotic susceptibility profiles of both strains are shown in Table 3.

All the in vitro experiments were performed in triplicate and on different days to ensure reproducibility.

The MIC results were interpreted according to the European Committee on Antimicrobial Susceptibility Testing (EUCAST 2024) guidelines [36], except for ceftazidime and cefepime, for which the Clinical and Laboratory Standards Institute (CLSI 2024) guidelines [37] were used. The breakpoint criteria based on the study reported are as follows. Amikacin: susceptible MIC ≤ 8 mg/L and resistant MIC > 8 mg/L; gentamycin: susceptible MIC ≤ 4 mg/L and resistant MIC > 4 mg/L; meropenem: susceptible MIC ≤ 2 mg/L and resistant MIC > 8 mg/L; ceftazidime: susceptible MIC ≤ 8 mg/L and resistant MIC ≥ 32 mg/L; cefepime: susceptible MIC ≤ 8 and resistant MIC ≥ 32 mg/L; sulbactam: susceptible MIC ≤ 4 mg/L, intermediate MIC = 8, and resistant MIC ≥ 16 mg/L [38]; colistin: susceptible MIC ≤ 2 mg/L and resistant MIC > 2 mg/L; ciprofloxacin: susceptible MIC ≤ 0.001 mg/L and resistant MIC > 1 mg/L; tigecycline: susceptible ≤ 0.5 mg/L and resistant > 0.5 mg/L [39]. Resistant strains are highlighted in bold.

### 4.3. Characterization of Test Strains

#### 4.3.1. Surface Motility Assay

Surface motility was measured as previously described [40]. Briefly, overnight cultures of each strain were adjusted in phosphate-buffered saline (Lonza, MD, USA) to an OD of 0.6 (600 nm). Then, a 3 μL drop of bacterial suspension was plated onto Luria–Bertani medium (Merck, Madrid, Spain) containing 0.3% agarose. The plates were incubated for 24 h at 37 °C with 80% humidity, and then, the surface extensions were measured. The studies were performed in triplicate on different days to ensure reproducibility.

#### 4.3.2. In Vitro Growth Curves and CIs 

The experiments were carried out in Mueller–Hinton Broth II (MHBII) (Merck, Madrid, Spain) with a starting inoculum of 5 × 10^5^ CFU/mL for each strain. Tubes were incubated at 37 °C, and samples were taken at 0, 2, 4, 8, and 24 h, serially diluted, and then plated on blood agar plates. Competitive growth between the two strains was assessed in MHBII by mixing 5 × 10^5^ CFU/mL of each strain in the same incubation tube. At the same time points detailed above, dilutions from these cultures were seeded on both blood agar and Mueller–Hinton agar plates containing 2 mg/L of colistin. Both strains grew on blood agar plates, and the colistin-resistant AbCR17 strain was also grown on the previously prepared colistin plates. CIs were defined as the number of recovered CFUs of AbCR17/the number of recovered CFUs of AbCS01, divided by the number of CFUs in the AbCR17 inoculum/the number of CFUs in the AbCS01 inoculum [41]. If no colonies were recovered, the limit of detection of the assay (1 CFU) was used. The studies were performed in triplicate on different days to ensure reproducibility.

#### 4.3.3. Biofilm Assay 

We performed the biofilm assay as previously described [42]. Briefly, the tested strains were cultured overnight at 160 rpm and 37 °C and then diluted to 5 × 10^5^ CFU/mL. A 200 μL volume of the suspension was added to a 96-well plate and grown overnight at 37 °C. Each well was washed and filled with 0.4% crystal violet (Merck, Madrid, Spain) and then incubated for 10 minutes. Next, the wells were washed again and filled with 96% ethanol. After 15 min at room temperature, biofilm formation was quantified by measuring the optical density (OD) at 580 nm (Asys UVM 340 Microplate Reader, Cambridge, UK). *A. baumannii* ACC001 (clinical strain isolated from Spanish hospitals during the GEIH-REIPI Spanish Multicenter *Acinetobacter baumannii* Study II 2000–2010, GenBank Umbrella project PRJNA422585) was used as the negative control, and *A. baumannii* ATCC 19,606 was used as the positive control. The studies were performed in triplicate on different days to ensure reproducibility.

### 4.4. In Vivo Studies

#### 4.4.1. Animals 

Immunocompetent male C57BL/6J mice, aged 7–9 weeks, were used in this study (Production and Experimentation Animal **Centre**, University of Seville, Seville, Spain). The mice had murine pathogen-free sanitary status and were assessed for genetic authenticity. This study was carried out following the recommendations of the Guide for the Care and Use of Laboratory Animals [43] and followed the 2010/63/EU directive on the protection of animals used for scientific research. The experiments were approved by the Committee on the Ethics of Animal Experiments (11-09-15-322) of the Ministerio de Agricultura, Pesca y Desarrollo Rural, Junta de Andalucia, Spain. 

#### 4.4.2. Single-Cell Preparations of Splenocytes

Single-cell preparations of splenocytes from C57BL/6J mice inoculated six weeks prior (Supplementary Materials) were prepared for each strain. First, the cells were enriched for memory B, CD4+ T, or CD8+ T lymphocytes using positive selection kits (Miltenyi Biotec; Madrid, Spain). Then, the cells were surface-stained directly ex vivo with combinations of anti-CD273/PE (Clone TY25), anti-CD80/APC (Clone 16-10A1), anti-IgD/FITC (Clone 11-26c.2a), anti-CD19/BV421 (Clone 6D5), anti-CD4/PE (Clone RM4-5), anti-CD44hi-APC (Clone IM7), and anti-CD8/PE-Cy7 (Clone 53-6.7) (all purchased from Biolegend, La Jolla, CA, USA) [44]. The cells were acquired using four-color flow cytometry using FACS Canto II at the Institute of Biomedicine of Seville flow cytometry facility, and the data were analyzed using FlowJo software (Tree Star).

#### 4.4.3. Adoptive Transfer of Memory Lymphocytes

Single-cell suspensions of spleen cells from male C57BL/6J mice previously inoculated intratracheally with each of the strains were counted in a non-lethal model of pneumonia (Supplementary Materials Table S1), and defined numbers of memory CD4+ T cells, CD8+ T cells, and donor B cells were injected iv in a volume of 0.2 mL. Briefly, anesthetized mice were inoculated intratracheally with 50 μL of non-lethal inoculums: 8.40 log_10_ CFU/mL and 8.98 log_10_ CFU/mL for AbCS01 and AbCR17, respectively. 

#### 4.4.4. Efficacy Studies in a Pneumonia Murine Model Infected with *A. baumannii* AbCS01 and *A. baumannii* AbCR17 Clinical Strains

An experimental pneumonia model previously characterized by our group was constructed [45]. Briefly, anesthetized C57BL/6J mice were intratracheally inoculated with the minimum lethal dose previously characterized for each strain: 9.90 log_10_ CFU/mL and 9.32 log_10_ CFU/mL for AbCS01 and AbCR17, respectively. Then, groups of 10 mice were randomly included in the following therapeutic groups: (i) controls (infected, untreated); (ii) tigecycline (5 mg/kg/bid/72 h subcutaneously (sc)); (iii) sulbactam (60 mg/kg/6h/72 h intramuscularly (im)); (iv) memory B lymphocytes (single dose iv); (v) memory CD4+ T lymphocytes (single dose iv); and (vi) memory CD8+ T lymphocytes (single dose iv). Antibiotic therapies started 4 h after inoculation and lasted 72 h. The dosages were selected based on their pharmacokinetics/pharmacodynamics (PK/PD) parameters and their proven efficacy in previous murine models of infection [29,32]. Single-cell preparations of memory B, CD4+ T, or CD8+ T cells were prepared on the same day as they were to be used for efficacy studies. The IV treatment with 2 × 10^6^ of each of the memory cells commenced 30 min after mice inoculation.

Immediately after animal death or sacrifice (sodium thiopental, intraperitoneally) at the end of this study, the animals were assessed as previously described for lung and blood concentration analyses and the determination of mortality rates [45].

### 4.5. Statistical Analysis

Bacterial concentrations are expressed as the mean ± standard deviation. Differences in bacterial concentrations between groups were compared using the Mann–Whitney U test. Survival rates are expressed as percentages and were compared among groups using the two-tailed Fisher’s test. A *p*-value of <0.05 was considered significant. SPSS v25.0 software was used to conduct the statistical analysis (SPSS Inc., Chicago, IL, USA).

## Figures and Tables

**Table 1:** Efficacy of adoptive transfer of memory lymphocytes in the experimental pneumonia model infected with multidrug-resistant (MDR) and colistin-susceptible *Acinetobacter baumannii* CS01.

Group	Dosage	*n*	Lung (Log_10_ CFU/g)	Blood (Log_10_ CFU/mL)	Survival (%)
CON	-	10	10.21 ± 0.23	9.10 ± 0.61	10
TGC	5 mg/kg/q12h/sc	10	8.23 ± 2.26 ^a^	5.23 ± 3.71 ^a^	40
SUL	60 mg/kg/q6h/im	10	6.29 ± 1.18 ^a^	1.19 ± 1.30 ^a,b^	90 ^a^
M-B	2 × 10^6^ cells/iv	10	5.59 ± 2.48 ^a,b^	1.81 ± 3.50 ^a,b^	80 ^a^
M-CD4+ T	2 × 10^6^ cells/iv	10	5.35 ± 2.47 ^a,b^	1.02 ± 2.49 ^a,b^	90 ^a^
M-CD8+ T	2 × 10^6^ cells/iv	10	6.64 ± 3.18 ^a^	3.35 ± 3.97 ^a^	60

CON: control; TGC: tigecycline; SUL: sulbactam; M-B: memory B lymphocytes; M-CD4+ T: memory CD4+ T lymphocytes; M-CD8+ T: memory CD8+ T lymphocytes; sc: subcutaneous; im: intramuscular; iv: intravenous; ^a^ *p* < 0.05, with respect to the CON group; ^b^ *p* < 0.05, with respect to the TGC group.

**Table 2:** Efficacy of adoptive transfer of memory lymphocytes in the experimental pneumonia model infected with MDR and colistin-resistant *A. baumannii* CR17.

Group	Dosage	*n*	Lung (Log_10_ CFU/g)	Blood (Log_10_ CFU/mL)	Survival (%)
CON	-	10	8.08 ± 2.56	5.84 ± 3.87	30
TGC	5 mg/kg/q12h/sc	9	7.96 ± 2.56	4.61 ± 4.24	50
SUL	60 mg/kg/q6h/im	10	5.19 ± 1.19 ^a,b,d^	1.19 ± 1.31 ^a^	90 ^a^
M-B	2 × 10^6^ cells/iv	10	3.63 ± 0.46 ^a,b,c,d^	0.67 ± 0.61 ^a^	100 ^a,b^
M-CD4+ T	2 × 10^6^ cells/iv	10	3.81 ± 0.81 ^a,b,c,d^	0.80 ± 0.92 ^a^	100 ^a,b^
M-CD8+ T	2 × 10^6^ cells/iv	10	7.67 ± 1.87	4.00 ± 3.52	70

CON: control; TGC: tigecycline; SUL: sulbactam; M-B: memory B lymphocytes; M-CD4+ T: memory CD4+ T lymphocytes; M-CD8+ T: memory CD8+ T lymphocytes; sc: subcutaneous; im: intramuscular; iv: intravenous; ^a^ *p* < 0.05, with respect to the CON group; ^b^ *p* < 0.05, with respect to the TGC group; ^c^ *p* < 0.05, with respect to the SUL group; ^d^ *p* < 0.05, with respect to the M-CD8+ T group.

**Table 3:** Minimal inhibitory concentration (MICs) and minimal bactericidal concentrations (MBCs) of different antibiotics for *A. baumannii* strains.

Antimicrobials	CS01		CR17	
	MIC (mg/L)	MBC (mg/L)	MIC (mg/L)	MBC (mg/L)
Amikacin	1	2	1	1
Gentamycin	2	2	≤0.50	0.50
Meropenem	**64**	**128**	**>256**	**>256**
Ceftazidime	**64**	**128**	**64**	**128**
Cefepime	**32**	**64**	8	**32**
Sulbactam	4	**8**	2	4
Colistin	≤0.50	≤0.50	**64**	**128**
Ciprofloxacin	**32**	**32**	**16**	**128**
Tigecycline	≤0.50	**2**	**4**	**8**

## Data Availability

The data presented in this study are available on request from the corresponding author.

## Supplementary Materials

The following supporting information can be downloaded at https://www.mdpi.com/article/10.3390/ijms251910550/s1.
