# Supplementary material for: Could the Adoptive Transfer of Memory Lymphocytes be an Alternative Treatment for Acinetobacter baumannii Infections?
Source: Int J Mol Sci. 2024 Sep 30;25(19):10550. doi: 10.3390/ijms251910550 (PMC11477510; doi:10.3390/ijms251910550)
Supplement: Supplementary file 1 [file ijms-25-10550-s001.zip › ijms-3229481-supplementary.pdf]

Could the adoptive transfer of memory lymphocytes be an alternative treatment for *Acinetobacter baumannii* infections?

Supplementary Data

Supplementary Materials and Methods

1. Characterization of non-lethal murine pneumonia model

To achieve with each of the study strains, the necessary inoculum capable of causing pulmonary and blood infection in the animal, without causing associated mortality (non-lethal pneumonia murine model), we inoculated intratracheally (50 µL) different dilutions of overnight MHB cultures for each strain. We started with an inoculum of 5 x 10^4 CFU/mL and up to the concentration needed to reach the desired target for each of the strains: 8.40 $\log_{10}$ CFU/mL and 8.98 $\log_{10}$ CFU/mL, for AbCS01 and AbCR17, respectively.

Then, groups of 20 mice were inoculated with those inoculums and two randomly selected mice were sacrificed (thiopental, ip) at the following time-points: 4 hours, 1-, 2-, 3-, 4-, 5-, 6-, 7-, 9-, and 14-days post-infection. Immediately after the sacrifice, lungs were aseptically extracted and processed for bacterial counts ($\log_{10}$ CFU/g) and qualitative blood infection (doi: 10.1016/j.jgar.2020.10.024). Bacterial concentration in lungs and bacteraemia rates are showed in Supplementary Table 1.

2. Efficacy studies in a pneumonia murine model infected with *A. baumannii* AbCS01 and *A. baumannii* AbCR17 clinical strains

Mice were anesthetized by an intraperitoneal injection of ketamine/diazepam. Trachea was cannulated with a blunt-tipped needle, and a 0.1 mL syringe (Hamilton Co., Reno, NV, USA)

was used for the inoculation of 50 µL of the minimum lethal dose previously characterized. The mice remained in a 30° position until awake of anesthesia.

To evaluate the efficacy, infected mice were randomly included in the following therapeutic groups (n = 10): i) controls (did not receive antimicrobial treatment); ii) tigecycline (5 mg/kg/bid subcutaneously); iii) sulbactam (60 mg/kg/6h intramuscularly); iv) memory B lymphocytes (single dose intravenous); v) memory CD4+ T lymphocytes (single dose intravenous); and vi) memory CD8+ T lymphocytes (single dose intravenous). The first dose of each antibiotic therapies was administered 4 hours post inoculation and lasted 72 hours. Memory B, CD4+ T, or CD8+ T cells were prepared freshly on the same day as they were used and the treatment started 30 minutes after mice inoculation. All animals were monitored for 72 hours.

Immediately after animal death or sacrifice at the end of the protocol (sodium thiopental, intraperitoneally), aseptic thoracotomy was performed. Quantitative culture was carried out for blood and lungs. Blood samples were collected by cardiac puncture. Then lungs were also removed on a sterile Petri dish, weighed, and homogenized in 2 mL of sterile saline solution 4 minutes (Stomacher, Tekmar Co., Cincinnati, OH, USA). Lung or blood samples were serially diluted, plated on blood agar plates and incubated at 37°C overnight. Results are expressed as mean ± standard deviation (log_{10} CFU/mL or log_{10} CFU/g in case of blood or lungs, respectively). For blood, qualitative studies were also carried out. Samples were inoculated in sterile 15 mL tubes with 2 mL of Müller-Hinton Broth (Merck, Madrid, Spain) and incubated at 37 °C overnight. Results of the blood cultures are expressed as positive or negative.

Supplementary Results

2.1. Characterization of Test Strains

2.1.1 Surface motility assay

After 24 hours of incubation, the diameter surface extensions of the studied strains were measured in centimeters. Both strains showed similar surface motility, the colistin-susceptible AbCS01 strain surface motility was 190 ± 0.06 mm and the colistin-resistant AbCR17 strain 210 ± 0.05 mm (Figure 1A).

2.1.2. In vitro growth curves and competition indices (CI).

At 2, 4, 8, and 24 hours post incubation competition index (CI) between AbCS01 and AbCR17 was assessed (Figure 1B). A CI = 0 indicates no competition between the two species; a CI > 0 indicates a competitive advantage for AbCR17, and CI < 0 indicates a competitive advantage for AbCS01. When grown alone, both bacterial strains showed similar growth at 24 h. The colistin-susceptible strain, AbCS01 strain showed a loss of fitness compared with the colistin-resistant AbCR17 strain (CI: 1.06, 0.49, and 0.51 at 2, 4, and 24 h, respectively).

Supplementary Table S1. Lungs bacterial concentration and bacteremia rate in a non-lethal pneumonia model by multidrug-resistant *A. baumannii* strains.

		Time post-infection									
**Strains**		**4h**	**1d**	**2d**	**3d**	**4d**	**5d**	**6d**	**7d**	**9d**	**14d**
**AbCS01**	**Log_{10} CFU/g ± SD**	8.5 ± 0.5	7.0 ± 0.2	3.0 ± 0.2	2.0 ± 0.3	2.1 ± 0.8	1.8 ± 0.5	1.6 ± 2.1	2.1 ± 0.2	1.1 ± 0.9	1.6 ± 0.2
	**Bacteraemia (%)**	100	50	0	0	0	50	0	100	0	0
**AbCR17**	**Log_{10} CFU/g ± SD**	6.7 ± 0.5	4.3 ± 1.5	3.2 ± 0.3	1.9 ± 0.1	2.0 ± 0.3	0.9 ± 1.3	1.2 ± 1.6	0.9 ± 1.3	1.1 ± 1.7	0.0 ± 0.0
	**Bacteraemia (%)**	100	50	100	100	100	100	100	0	50	0

Bacterial concentration expressed as mean ± standard deviation (SD); h: hours; d: days.
